# Effects of Sodium Hypochlorite on Corrosion Behavior and Cyclic Fatigue in Different Types of NiTi Rotary Endodontic Instruments

**DOI:** 10.3390/ma19132818

**Published:** 2026-07-02

**Authors:** Nenad M. Stošić, Jelena Z. Popović, Dušan Petković, Aleksandar Mitić, Kosta Todorović, Marija Nikolić, Radomir Barac, Antonije Stanković, Milan Miljković, Milica S. Petrović, Milan Spasić, Ana Todorović

**Affiliations:** 1 Department of Restorative Dentistry and Endodontics, Clinic of Dental Medicine, Faculty of Medicine, University of Niš, 18000 Niš, Serbia; jelena.popovic@medfak.ni.ac.rs (J.Z.P.); aleksandar.mitic@medfak.ni.ac.rs (A.M.); marija.nikolic@medfak.ni.ac.rs (M.N.); radomir.barac@medfak.ni.ac.rs (R.B.); antonije.stankovic@medfak.ni.ac.rs (A.S.); milan.miljkovic@medfak.ni.ac.rs (M.M.); 2 Department of Production and IT Technologies, Faculty of Mechanical Engineering, University of Niš, 18000 Niš, Serbia; dusan.petkovic@masfak.ni.ac.rs; 3 Department of Oral Surgery, Clinic of Dental Medicine, Faculty of Medicine, University of Niš, 18000 Niš, Serbia; kosta.todorovic@medfak.ni.ac.rs (K.T.); milan.spasic@medfak.ni.ac.rs (M.S.); 4 Department of Oral Medicine and Periodontology, Clinic of Dental Medicine, Faculty of Medicine, University of Niš, 18000 Niš, Serbia; petrovicsmilica@gmail.com; 5 Department of Orthodontics, Clinic of Dental Medicine, Faculty of Medicine, University of Niš, 18000 Niš, Serbia; ana.todorovic@medfak.ni.ac.rs

**Keywords:** rotary endodontic instruments, nickel titanium, sodium hypochlorite, corrosion, cyclic fatigue

## Abstract

Sodium hypochlorite (NaOCl), as the most commonly used irrigant solution, offers strong antibacterial and organic tissue dissolving properties and has the potential to express a strong corrosive effect. The aim of the study was to evaluate the effects of NaOCl on corrosion behavior and cyclic fatigue in five different types of NiTi rotary endodontic instruments. The study examined: ProTaper Universal, ProTaper Next, Twisted File, HyFlex CM, and BioRace, each with 24 samples. Each group was divided into a control subgroup and a subgroup exposed to electrochemical testing of corrosion in 5.25% NaOCl. All instruments were exposed to a cyclic fatigue test in artificial canals with a curvature angle of 60 degrees and radius of 2 mm. The corrosion potential of NaOCl was determined in all groups of instruments. ProTaper Universal had a significantly lower corrosion resistance than ProTaper Next (*p* < 0.05), Twisted File, BioRace, and HyFlex CM (*p* < 0.001). HyFlex CM showed significantly higher resistance to cyclic fatigue compared to all the other groups of instruments (*p* < 0.001). Surface corrosion reduced cyclic fatigue resistance in most of the instrument groups, except in heat-treated instruments with controlled memory. HyFlex CM has proven to have the highest resistance to corrosion and cyclic fatigue.

## 1. Introduction

Endodontic instruments are used daily in tooth root canal treatment in order to treat pathologies of dental pulp or periapical tissues. The purpose of every endodontic treatment is to perform a good mechanical preparation of the root canals of the affected tooth. This includes treating the prepared root canal system with disinfectants and medications in order to prepare it for the final obturation [1,2].

Considering the three-dimensional complexity of root canal morphology in most teeth, the outcome of the treatment depends to a large extent on the quality of the used endodontic instruments. Therefore, it is of great importance to select instruments that will not undergo chemical change or deformation during the therapeutic procedure [3].

Rotary nickel–titanium (NiTi) endodontic instruments have largely replaced hand instruments in everyday practice, primarily due to their high flexibility and cutting efficiency. These features enable faster, more controlled, and anatomically precise root canal preparation [3,4]. During clinical use, NiTi instruments are exposed to two dominant types of mechanical loading: cyclic fatigue, which occurs due to repeated bending in curved canals, and torsional stress, which occurs when engaging the dentine walls [5]. Literature data show that cyclic fatigue is the most common cause of rotary instrument fracture [5]. This kind of fracture occurs suddenly and without previous visible deformations, which leads to complications in further treatment [6]. Due to its frequency and clinical relevance, cyclic fatigue represents one of the most intensively researched topics in endodontic science, along with the development of NiTi alloys and the improvement of surface treatment technologies [7]. By introducing rotary NiTi instruments, it is possible to overcome the problem of sudden fracture due to their high elasticity, shape memory effect, and biocompatibility. These features are a result of the specific thermoelastic martensitic transformation of the alloys that is influenced by their mechanical and thermal treatment [8,9,10]. Thermal treatment and the surface conditioning of NiTi alloys have a significant effect on their mechanical properties and resistance to corrosion. Heat treatment, twisting, and electropolishing are some of the thermomechanical processing methods that can modify oxide layer thickness and nickel ion release and increase the alloy’s resistance to corrosion and cyclic fatigue [10,11,12,13].

An essential part of endodontic therapy during the mechanical preparation of the tooth root canal is extensive irrigation with specific chemical disinfectant solutions [14]. The most commonly used irrigant for this purpose, sodium hypochlorite (NaOCl), in addition to its strong antibacterial and organic tissue dissolving properties, has a significant corrosive effect due to the presence of aggressive chloride anions [15,16]. These ions selectively remove nickel from the surface of instruments, which can lead to microstructural defects that represent weak points and centers of stress accumulation. They in turn lead to the occurrence of early fractures during mechanical preparation of the root canal [17]. Literature data have shown that corrosion is one of the first factors that promotes fatigue of the materials that endodontic instruments are made of, rendering them more susceptible to deformation and fracture in further use [18]. Studies of the effects of electrochemical behavior of differently treated NiTi alloys in a potential corrosive environment represent a prerequisite for producing more durable and safer endodontic instruments [19,20,21]. Therefore, it is important to investigate the occurrence of corrosion in instruments made of different alloys and subjected to a different surface and thermal treatment in a NaOCl solution, which contributes to fatigue and fracture of the instruments [22].

The aim of the study was to evaluate the influence of NaOCl on corrosion behavior in five different types of NiTi rotary endodontic instruments and to compare the resistance to cyclic fatigue of the instruments subjected to various production technologies.

## 2. Materials and Methods

### 2.1. Sample Size Calculation

An a priori sample size calculation was performed using G*Power software (version 3.1.9.7; Heinrich Heine University Düsseldorf, Düsseldorf, Germany). The calculation was based on previously published data from a comparable in vitro study by Nogueira et al. [21], which investigated the effect of sodium hypochlorite immersion on the cyclic fatigue resistance of NiTi rotary instruments using the number of cycles to fracture (NCF) as the primary outcome. Assuming a two-tailed independent-samples *t*-test, a significance level (α) of 0.05, a statistical power of 80%, and an allocation ratio of 1:1, the minimum required sample size was estimated to be five specimens per subgroup. Therefore, the inclusion of 12 specimens per subgroup (24 specimens per instrument system) was considered sufficient to detect statistically significant differences in cyclic fatigue resistance.

### 2.2. Endodontic Instruments Used in the Research

A sample of 120 instruments was included in this study. Five types of NiTi rotary instruments, one conventional and four new-generation treated types, were analyzed. Each type of instrument presented a separate group that included 24 samples (Table 1). Each group was divided into two subgroups: the first subgroup consisted of 12 instruments that were used as a control (non-corroded), while the second subgroup consisted of 12 instruments that were exposed to the electrochemical process. Both groups were exposed to a cyclic fatigue test.

### 2.3. Electrochemical Testing

A total of 60 instruments, twelve of each type, were tested for corrosion sensitivity. Measurements were performed in a 5.25% NaOCl solution (Chloraxid 5.25% Cerkamed Medical Company, Stalowa Wola, Poland). The potentiodynamic electrochemical test methodology was used to evaluate the corrosion behavior of the instruments. Tests were performed by using a one-piece corrosion cell of 1 Potentiostat/Galvanostat PGSTAT 128N with the appropriate software. A platinum spiral was used as a counter electrode. All the potentials were measured relative to a reference silver–silver chloride electrode (Ag/AgCl). The working electrode, in this case the working part of the tested endodontic instrument, was immersed in a NaOCl solution which was freshly poured every time into the corrosion cell before the start of the measurement. The working part of each instrument was immersed in the solution, while the flat part intended for fastening/holding was not. The software was set for the instruments to be immersed in the solution for 15 s before the start of the potential rise. The initial potential was chosen to be 0.1–0.2 V lower than the open circuit potential. Recording of anodic polarization curves E and I was performed by using the appropriate NOVA 2.1.5 software, specifically the technique of linear increase in potential with a speed of 0.2 mV/s. The temperature of the solution was ambient (23 ± 3 °C). The value of the potential, at which there is a sudden increase in the current, is marked as the pitting potential (Up). The sudden increase in the current density is the result of the local dissolution of the metal surface and the formation of pits in those places. Measurements were performed on twelve samples of each instrument type subgroup. The corrosion resistance results represented the mean value of pitting potential for the samples of each experimental group. After the electrochemical test, the instruments were subjected to a cyclic fatigue test.

### 2.4. Cyclic Fatigue Testing

Cyclic fatigue testing was performed on 24 samples in each experimental group. The impact of corrosion damage on the structure of the instruments was analyzed on twelve samples from each subgroup that was exposed to the electrochemical process, while the other twelve instruments that were not exposed to the electrochemical process represented the control.

A unique stainless steel metal block was made in accordance with the research of Plotino et al. [23] to facilitate the experiment. The block included an artificial canal 1.4 mm in diameter, which was machine cut to a 60-degree angle of curvature for instrument testing, with a 2 mm curvature radius. Tempered glass was placed over the canal to prevent the broken piece from falling out, as well as to allow inspection of the rotating device. A metal ring holder perpendicular to the canal held the handpiece in place. Using an electric endo motor (X-Smart Plus, Dentsply Sirona, Ballaigues, Switzerland), the instruments were continuously rotated to the right upon entering, all the way to the top of the canal. Rotation speed and resistance were applied according to the manufacturer’s recommendations (ProTaper Universal—250 rpm, 2.5 Ncm torque; ProTaper Next—300 rpm, 2.0 Ncm torque; Twisted File—500 rpm, 2.0 Ncm torque; HyFlex CM—500 rpm, 2.5 Ncm torque; BioRace—600 rpm, 1.0 Ncm torque). The friction of the instruments with stainless steel walls was reduced by the use of glycerin. The rotation time up to the moment of the fragment’s breakage, recorded audibly and visually, was measured with a digital stopwatch. Cyclic fatigue is defined by the number of cycles to fracture value (NCF).

$$NCF=\frac{rotational\;speed\;of\;the\;instrument\times time\;to\;fracture\;in\;seconds}{60}$$

### 2.5. Statistical Analysis

Statistical data analysis was performed by applying appropriate parametric tests in the IBM SPSS program version 26.0, with a *p* < 0.05 degree of probability. Analysis of variance (ANOVA) was used to determine the existence of differences in the values of electric potential between the examined groups, while a Tukey post hoc test was applied to identify the exact pairs of groups between which these differences were statistically significant.

The Student’s *t*-test for independent samples was used to compare NCF values between treated and non-treated instruments. In addition, analysis of variance (ANOVA) was applied for the comparative assessment of NCF values in the groups of treated and non-treated instruments, after which the Tukey post hoc test was used to accurately determine differences between individual groups of instruments.

### 2.6. SEM Analysis

After the cyclic fatigue test, the fractured instrument fragments were ultrasonically cleaned in 70% alcohol (Sonic 4G, 40 KHz, Sonic, Niš, Serbia) to prepare them for ultrastructural analysis. Following this, the samples were mounted on aluminum cylindrical stubs using an adhesive (Dotite paint xc 12 Carbon JEOL, Tokyo, Japan), vacuumed, and coated with a layer of gold in an ion sputtering device (JFC 1100E Ion Sputter JEOL, Tokyo, Japan). After preparation, the fragments were observed and evaluated using a JEOL-JSM-5300 scanning electron microscope (JEOL Ltd., Tokio, Japan) at magnifications of 100×, 200×, and 2000×.

## 3. Results

### 3.1. Electrochemical Analysis

**Figure 1:**
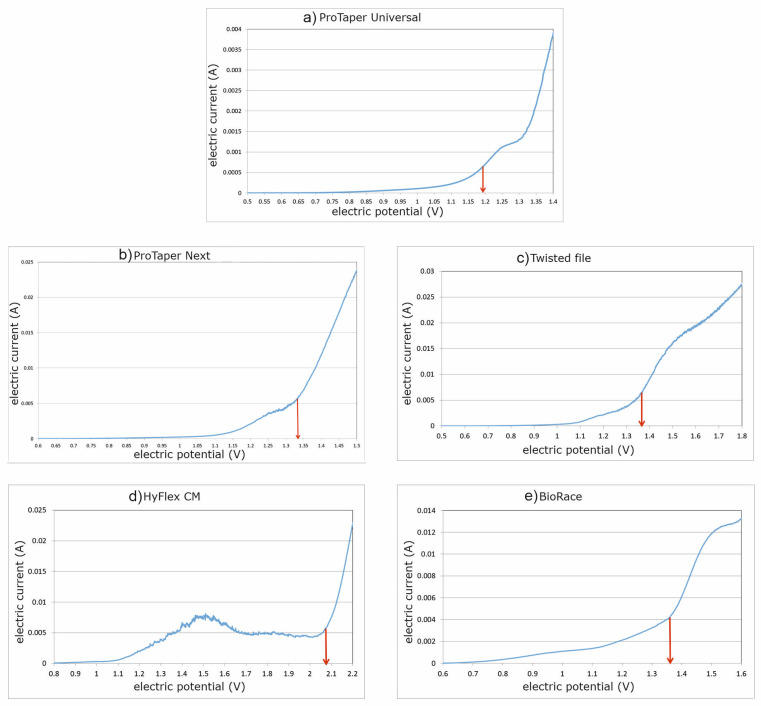
Potentiodynamic polarization curve of the tested instruments: (**a**) ProTaper Universal, (**b**) ProTaper Next, (**c**) Twisted File, (**d**) HyFlex CM, (**e**) BioRace in 5.25% NaOCl.

Electrochemical testing revealed that all tested NiTi rotary instruments demonstrated susceptibility to corrosion after exposure to 5.25% NaOCl (Figure 1). The lowest corrosion resistance was observed in the ProTaper Universal group, with a pitting potential of 1.27 V. ProTaper Next showed slightly greater resistance (1.33 V), followed by Twisted File (1.36 V), and BioRace (1.37 V). HyFlex CM exhibited the highest corrosion resistance, with a significantly higher pitting potential of 2.08 V.

The ANOVA test confirmed the existence of differences in electrochemical pitting potential values between the tested groups (*p* < 0.001). The Tukey post hoc analysis showed that the electrochemical potential of the ProTaper Universal subgroup of instruments was significantly lower compared to the Twisted File, HyFlex CM, and BioRace subgroups (*p* < 0.001), as well as ProTaper Next (*p* < 0.05). On the other hand, in the HyFlex CM subgroup of instruments, the electrochemical potential was significantly higher than for all the other instruments (*p* < 0.001) (Table 2).

### 3.2. Cyclic Fatigue Analysis

Among the control (non-corroded) subgroup of instruments, ProTaper Universal showed the lowest cyclic fatigue resistance, with significantly lower NCF values than all other instrument subgroups (*p* < 0.001). Conversely, the HyFlex CM subgroup of instruments exhibited a significantly higher number of rotations to fracture than all the other tested subgroups (*p* < 0.001). Furthermore, the NCF values of the ProTaper Next were significantly higher than those of the BioRace (*p* < 0.001) and Twisted File (*p* < 0.001) subgroups of instruments. Moreover, the NCF values of the instruments belonging to the Twisted File subgroup were notably higher than those of the BioRace subgroup (*p* < 0.001) (Table 3).

Upon exposure of the experimental subgroups to electrochemical testing and follow-up cyclic fatigue testing, a significantly lower NCF was observed in the ProTaper Universal subgroup of instruments than in all the other subgroups (*p* < 0.001). Specifically, the instruments of the HyFlex CM subgroup showed the highest NCF (*p* < 0.001), while the NCF values of the BioRace were much lower compared to the ProTaper Next (*p* < 0.001) and Twisted File subgroups (*p* < 0.001) (Table 3).

A significant reduction in cyclic fatigue resistance after electrochemical testing was observed in the ProTaper Universal (*p* < 0.001), ProTaper Next (*p* < 0.001), and Twisted File (*p* < 0.001) instrument groups. Although HyFlex CM instruments showed an increase in NCF, while BioRace instruments showed a decrease, in both cases the differences were not statistically significant (Table 3).

**Figure 2:**
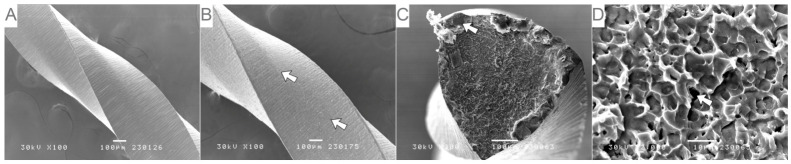
Scanning electron microscopy of the ProTaper Universal control (non-corroded) subgroup (**A**) at magnifications of 100× and subgroup upon exposure to electrochemical testing (**B**–**D**) and follow-up cyclic fatigue testing: (**B**) the active working part of the instrument (100× magnification) showing surface microdamage and signs of localized corrosion in the form of pits (white arrow), (**C**) a cross-section (200× magnification) with characteristic convex triangular geometry for a ProTaper Universal instrument, as well as traces of deformation and initial cracks caused by cyclic fatigue (white arrow), (**D**) microstructural analysis at higher magnification (2000×) of the ductile surface of the fractured fragment with characteristic relief, pits, and cracks caused by the fracture of the NiTi alloy during cyclic fatigue (white arrow).

**Figure 3:**
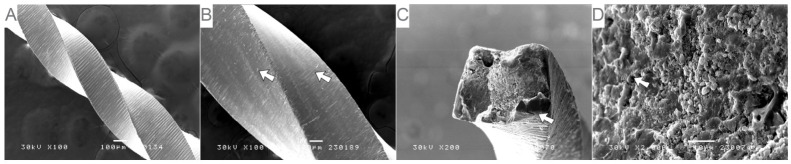
Scanning electron microscopy of the ProTaper Next control (non-corroded) subgroup (**A**) at magnification of 100× and subgroup upon exposure to electrochemical testing (**B**–**D**) and follow-up cyclic fatigue testing: (**B**) the external working part of the instrument (100× magnification), examining the surface with signs of pitting corrosion (white arrow), (**C**) a rectangular cross-section (200× magnification) for a ProTaper Next instrument, with deformations on the periphery caused by cyclic fatigue (white arrow), (**D**) a microstructural view at higher magnification (2000×) showing the ductile surface of the fractured fragment with microvoids or pits caused by NiTi ruptures (white arrow).

**Figure 4:**
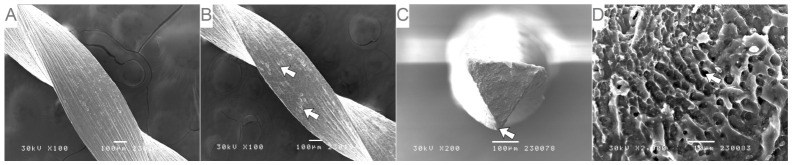
Scanning electron microscopy of the Twisted File control (non-corroded) subgroup (**A**) at magnification of 100× and subgroup upon exposure to electrochemical testing (**B**–**D**) and follow-up cyclic fatigue testing: (**B**) the lateral side of the active working part of the instrument (100× magnification) demonstrating localized corrosion in pit formation (white arrow), (**C**) a cross-section aspect of a Twisted File instrument (200× magnification) with a typically triangular form and initial fatigue cracks (white arrow), (**D**) microstructural observation at higher magnification (2000×) presenting a pliant pattern with characteristic dimples and microvoids (white arrow).

**Figure 5:**
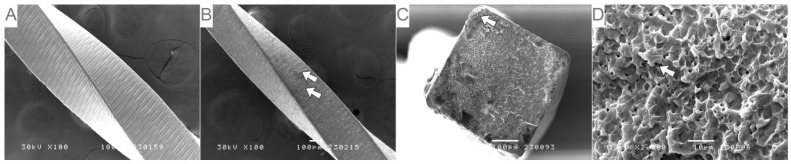
Scanning electron microscopy of the HyFlex CM control (non-corroded) subgroup (**A**) at magnification of 100× and subgroup upon exposure to electrochemical testing (**B**–**D**) and follow-up cyclic fatigue testing: (**B**) the external lateral surface of a HyFlex CM instrument (100× magnification) with visible microdamage and signs of localized corrosion pits (white arrow), (**C**) a cross-section (200× magnification) with characteristic quadrangular geometry, as well as traces of deformation and initial cracks caused by cyclic fatigue (white arrow), (**D**) higher magnification microstructural evaluation (2000×) presenting a ductile fractured surface illustrating deep dimples and striations caused by the fracture of the NiTi alloy (white arrow).

**Figure 6:**
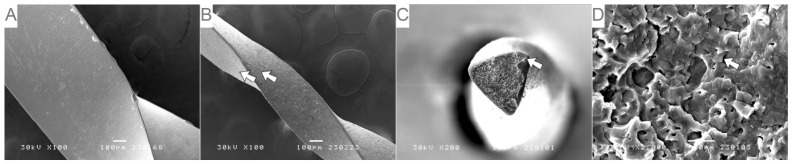
Scanning electron microscopy of the BioRace control (non-corroded) subgroup (**A**) at magnification of 100× and subgroup upon exposure to electrochemical testing (**B**–**D**) and follow-up cyclic fatigue testing: (**B**) the external instrument profile (100× magnification) exhibiting a surface with microdamage and localized corrosion in the form of pits (white arrow), (**C**) a triangular cross-section (200× magnification) of a BioRace instrument, with visible initial fatigue cracks (white arrow), (**D**) a micrograph at 2000× demonstrating the ductile surface of the fractured fragment with microvoids or pits caused by NiTi ruptures (white arrow).

Figure 2, Figure 3, Figure 4, Figure 5 and Figure 6 show SEM images of the fractured part of the tested instruments, control (non-corroded) subgroup at magnifications of 100× and subgroup after exposure to electrochemical testing and cyclic fatigue at magnifications of 100×, 200×, and 2000×. In all tested instrument subgroups, the SEM analysis showed characteristic signs of material degradation in the form of localized corrosion spots and initial cracks. Additionally, the microstructure itself revealed areas of ductile fracture, striations, microvoids, and deep dimples.

## 4. Discussion

Endodontic treatment of an infected tooth’s root canal cannot adequately be performed without proper chemomechanical treatment of the canal system. In clinical practice, the cumulative action among active Cl- ions from a NaOCl solution and the metal surface of NiTi rotary endodontic instruments induces localized corrosion, which damages the instrument’s protective layer and compromises the integrity of the instrument’s material [16].

After exposure of the instruments to 5.25% NaOCl, increased current and corrosion tendency were observed. In instruments where NaOCl caused a greater increase in the current, a decrease in corrosion resistance was indicated. Due to the high current density during electrochemical testing, localized corrosion microdefects appeared, which led to a decrease in the material’s resistance to cyclic fatigue. According to that mechanism, corrosion resistance was lowest for ProTaper Universal instruments. Corrosion resistance increased in the following order: ProTaper Next, Twisted File, BioRace, and HyFlex CM.

The obtained results are in a positive correlation with data available in the literature [17,24]. The research has determined that HyFlex CM has greater corrosion resistance, not only compared to conventional instruments but also to the other three thermally treated instrument types.

Corrosion resistance could be dependent on the manufacturing process and on the surface treatment applied to the rotating instrument’s working parts [1]. The primary advantage of thermomechanical treatments of NiTi alloy is reflected in their ability to modify the transformation temperatures of a phase. The phases vary from austenite, the R-phase, and martensite, resulting in a visible improvement of the alloy’s mechanical properties [25,26]. One of the indicators suggesting that the instruments are subjected to thermomechanical treatments are the specific color and thickness of the titanium dioxide layer formed on the working part of the instrument [27]. Given that, according to some authors, the corrosion resistance of a rotating instrument depends on the presence and thickness of a passive titanium oxide layer [28,29,30]. The expression of these characteristics correlates with the thermal protocols to which the instruments were exposed. Each of these treatments induces modifications in the performance of the instrument in terms of increasing flexibility, cyclic fatigue resistance, and resistance to the aggressive effects of certain irrigants. This in turn contributes to their efficiency and safer use in clinical practice [27].

It should be emphasized that ProTaper Universal instruments showed the highest sensitivity to corrosion, which was reflected in the lower NCF values compared to the control. The literature indicates that immersing ProTaper Universal and ProTaper Next instruments in a NaOCl solution led to an electrolytic reaction with the metal surface and the appearance of visible dark particles on the surface of the solution and subsequent corrosion [31]. These results are consistent with the findings of the present study and those of other authors [32,33]. Oktavia et al. investigated the effect of NaOCl and EDTA in two types of rotating NiTi instruments and determined that the occurrence of corrosion sensitivity and surface damage was significantly higher in ProTaper Universal [32]. However, some studies showed that NaOCl did not reduce the cyclic fatigue of NiTi rotary instruments [1,34,35]. The results of this study were in contrast to the results of Li et al., which showed surprising resistance of the ProTaper Universal in relation to three of the four analyzed instruments [1]. Due to the use of classical cutting technology [36], the ProTaper Universal instrument has traces of processing on the surface, as well as microdefects which are particularly sensitive places for the manifestation of the corrosive effect of irrigation solutions [37]. Also, these types of instruments are entirely made of an austenitic structure, which makes them more rigid than martensitic ones [37].

Higher corrosion resistance of rotary ProTaper Next instruments could be explained by modifications in the fabrication of NiTi alloys and the improved microstructure of the material as a result of the heat treatment process [3]. These instruments operate predominantly in the martensite phase, which is more elastic and softer, leading to stress decrease within the material and significantly better behavior in the presence of corrosive NaOCl [3]. Owing to the continuous improvement of technology based on M-wire technology and the exposure of NiTi wire to heat treatments, with a higher proportion of the martensite phase, ProTaper Next is more resistant than the conventional ProTaper Universal instrument [36]. Abuhaimed [38] researched the effects of several different solutions on corrosion occurrence and found a significant effect of NaOCl on both corrosion behavior and NCF reduction in ProTaper Next, which is also in accordance with the findings from existing literature [21,37].

The results of this study showed that Twisted File proved to be more resistant compared to ProTaper Universal. As a result of the manufacturing method, which is reflected in the specific heat treatment that provides the formation of a passive titanium oxide layer, followed by the twisting technology, the R-phase is obtained which preserves the continuity of the metal fibers [39]. This allows the preservation of the natural grain structure, which results in a lower rate of crack formation and their slow propagation [40]. An increased corrosion resistance is usually observed in the presence of oxide layers of NiTi alloys in an extremely aggressive environment such as NaOCl, which is why an increase in corrosion resistance is expected in Twisted File compared to conventional ProTaper Universal instruments [41]. The results of this research are in accordance with numerous studies that dealt with the mentioned issue [20,42,43,44]. Pedula et al. examined the resistance to cyclic fatigue of thermally treated NiTi instruments during static or dynamic immersion in a 5% NaOCl solution, which led to a decrease in the mean values of NCF in Twisted File instruments [20]. However, literature data showed that the NaOCl solution did not affect the reduction of the cyclic fatigue resistance of this instrument [45].

Another method for finishing endodontic instruments to enhance their properties, such as cutting efficiency, greater resistance to cyclic fatigue, and corrosion, is electropolishing. The essence of this process is the removal of the elemental Ni and leaving a homogeneous oxide layer. Such a procedure leaves minimal chances for the occurrence of surface defects as potential stress centers [17,46,47]. This procedure largely removes irregularities that are inherent in conventional instruments and which may have serious consequences leading to surface corrosion defects and fractures. The results of this study showed that the resistance of BioRace instruments to corrosion and cyclic fatigue after exposure to a NaOCl solution was minimally reduced, demonstrating their resistance to corrosive effects. The results of this research are in accordance with the results of other studies [17,32]. In the research of Medojevic et al., who tested the effect of NaOCl on the corrosion of five different endodontic NiTi instruments, the BioRace rotary endodontic instrument demonstrated the highest resistance to corrosion and cyclic fatigue [17]. In contrast, previous studies observed higher corrosion resistance in conventional instruments compared to electropolished ones [1,48,49]. Similarly, Topçuoglu et al. [35] reported no advantage in cyclic fatigue resistance and corrosion resistance for electropolished BioRace over conventional ProTaper Universal and Mtwo following NaOCl exposure.

The results of this study showed that HyFlex CM instruments, after electrochemical testing, exhibited statistically significantly higher resistance to cyclic fatigue compared to instruments from other experimental groups. A significant finding of this study is that the NCF value for HyFlex CM after exposure to the corrosive effect of NaOCl was higher than that of the control non-corroded group. The high values of the pitting potential of HyFlex CM, which were also the highest compared to other instruments, could indicate the presence of a more stable TiO_2_ layer that affects the reduced susceptibility to localized corrosion [50]. The specificity of the material from which HyFlex CM is made, reflected in its prominent hardness and the ability to return to its original shape after heat exposure, is the memory effect. Also, the predominance of the martensitic phase in HyFlex CM affects the increase in flexibility and improved resistance to crack propagation [13,51]. This could be the reason why the NCF after the corrosion resistance test remained high. As previously mentioned, corrosion implies the selective removal of nickel from the surface of the instrument, which results in the formation of microstructural surface defects. The specificity of the composition of the alloy, especially the nickel content, which is lower in the HyFlex CM compared to the other rotating systems and amounts to 52% of the weight, could play a role in significant resistance to corrosion and high resistance to cyclic fatigue. Although elemental analysis (EDS/XPC), nickel release measurements and surface oxide analysis were not directly performed in this study, previous research suggests that metallurgical properties might explain the observed significant resistance to corrosion and cyclic fatigue [44,52]. Literature data show contradictory findings regarding the testing of HyFlex CM instruments’ susceptibility to corrosion [21,53]. In the research of Dadgar et al. [53], the effect of NaOCl on the resistance to cyclic fatigue of three different rotating instruments, RaCe, HyFlex CM, and XP-endo shaper files, was evaluated. The research proved the significant influence of NaOCl on the appearance of corrosion and thus on the appearance of a decrease in resistance to cyclic fatigue. In contrast, Nair et al. analyzed the cyclic fatigue resistance of Twisted File, HyFlex CM, HyFlex EDM, and Edgefile X3 EDM after immersion in a NaOCl solution and reported that dynamic immersion in 2.5% NaOCl for a longer period positively affected the resistance of the HyFlex CM rotary instrument to cyclic fatigue. This finding was in accordance with the results of our study [43].

The limitation of the study primarily relates to the difference in the conditions of instrument application in clinical conditions. Most of these laboratory experiments are conducted at room temperature, where there is a difference in humidity and temperature compared to the conditions in the oral cavity, which can affect the corrosion susceptibility of the instruments and resistance to cyclic fatigue. Additionally, a limitation of this study is that the evaluated rotary NiTi endodontic instruments differ in their cross-sectional geometry, tapers, core diameter and rotational speeds, meaning the observed differences reflect the cumulative effect of each system’s complete design rather than the isolated impact of NaOCI corrosion alone. These factors are known to significantly affect both cyclic fatigue resistance and mechanical performance. This study, like most other studies, was conducted on a static model that tests instruments for cyclic fatigue while excluding the effects of torsional stress, and it is possible that different results could be obtained under clinical conditions.

## 5. Conclusions

The corrosive effect of NaOCl has been proven on all five types of instruments. The highest resistance to the corrosive effect of NaOCl was observed in the HyFlex CM instruments. Surface corrosion reduced cyclic fatigue resistance in most of the instrument groups, except in heat-treated instruments with controlled memory. The lowest resistance to fracture was in the conventional instrument ProTaper Universal, while the HyFlex CM instrument showed the highest resistance to cyclic fatigue after corrosion.

## Figures and Tables

**Table 1:** Instruments used in the research.

Group	File	ISO Size	Taper	Cross Section	Manufacturer	Production Technology	Heat Treatment	Alloy
I	ProTaper Universal	25	0.04	Convex triangular	Dentsply Sirona, Ballaigues, Switzerland	Conventional instrument	No	55.8% Ni, 44.2% Ti
II	ProTaper Next	25	0.06	Rectangular	Dentsply Sirona, Ballaigues, Switzerland	M—wire technology	Yes	55.8% Ni, 44.2% Ti
III	Twisted File	25	0.04	Triangular	SybronEndo, Orange, CA, USA	R—wire technology	Yes	55.2% Ni, 44.8% Ti
IV	HyFlex CM	25	0.04	Quadrangular	Coltene, Whaledent, Altstätten, Switzerland	Thermally processed instruments	Yes	52% Ni, 48% Ti
V	BioRace	25	0.04	Triangular	FKG, Dentaire, Switzerland	Electropolished instrument	No	54.5% Ni, 45.5% Ti

**Table 2:** The Tukey Post hoc Test: Multiple comparisons of electrochemical potential between instruments.

	ProTaper Universal	ProTaper Next	Twisted File	HyFlex CM	BioRace
ProTaper Universal	/	*p* = 0.006 **	*p* < 0.001 *	*p* < 0.001 *	*p* < 0.001 *
ProTaper Next	*p* = 0.006 **	/	*p* = 0.651	*p* < 0.001 *	*p* = 0.204
Twisted File	*p* < 0.001 *	*p* = 0.651	/	*p* < 0.001 *	*p* = 0.903
HyFlex CM	*p* < 0.001 *	*p* < 0.001 *	*p* < 0.001 *	/	*p* < 0.001 *
BioRace	*p* < 0.001 *	*p* = 0.204	*p* = 0.903	*p* < 0.001 *	/

*p*—degree of probability, *—significant at *p* < 0.001, **—significant at *p* < 0.05.

**Table 3:** Arithmetic mean ($\bar{x}$) ± standard deviation (SD) of NCF value obtained when testing instruments in a canal with a curvature angle of 60º and a radius of curvature of 2 mm before and after electrochemical testing.

	ProTaper Universal	ProTaper Next	Twisted File	HyFlex CM	BioRace	ANOVA	Tukey Post Hoc Test *p* < 0.001 *
Control (non-corroded)	226.25 ± 3.72	893.17 ± 6.22	846.33 ± 4.58	4896.25 ± 7.97	542.24 ± 5.01	*p* < 0.001 *	1 vs. 2, 1 vs. 3, 1 vs. 4, 1 vs. 5, 2 vs. 3, 2 vs. 4, 2 vs. 5, 3 vs. 4, 3 vs. 5, 4 vs. 5
Electrochemically tested	174.25 ± 5.78	718.42 ± 32.28	766.17 ± 4.17	4920.08 ± 148.94	538.08 ± 18.47	*p* < 0.001 *	1 vs. 2, 1 vs. 3, 1 vs. 4, 1 vs. 5, 2 vs. 4, 2 vs. 5, 3 vs. 4, 3 vs. 5, 4 vs. 5
Student’s *t*-test	*p* < 0.001 *	*p* < 0.001 *	*p* < 0.001 *	*p* = 0.59	*p* = 0.46		

*p*—degree of probability, *—significant at *p* < 0.001.

## Data Availability

The original contributions presented in this study are included in the article. Further inquiries can be directed to the corresponding author.

## Abbreviations

The following abbreviations are used in this manuscript:

rpm	Rotations per minute
Ncm	Newton-centimeter
NCF	Number of cycles to fracture value
SEM	Scanning electron microscopy
NaOCl	Sodium hypochlorite
